# Quantitative evaluation of biomechanical properties of optic nerve head by using acoustic radiation force optical coherence elastography

**DOI:** 10.1117/1.NPh.10.4.045008

**Published:** 2023-12-05

**Authors:** Gang Shi, Yubao Zhang, Xiao Han, Sizhu Ai, Yidi Wang, Yingji Li, Jiulin Shi, Xingdao He, Xinhe Zheng

**Affiliations:** a University of Science and Technology Beijing, School of Mathematics and Physics, Beijing Key Laboratory for Magneto-Photoelectrical Composite and Interface Science, Beijing, China; b Nanchang Hangkong University, Key Laboratory of Opto-Electronic Information Science and Technology of Jiangxi Province, Jiangxi Engineering Laboratory for Optoelectronics Testing Technology, Nanchang, China

**Keywords:** optical coherence tomography, optical coherence elastography, optic never head, biomechanical properties, phase velocity

## Abstract

**Significance:**

Previous studies have demonstrated that the biomechanical properties of the optic nerve head (ONH) are associated with a variety of ophthalmic diseases; however, they have not been adequately studied.

**Aim:**

We aimed to obtain a two-dimensional (2D) velocity distribution image based on the one-to-one correspondence between velocity values and position using the acoustic radiation force optical coherence elastography (ARF-OCE) technique combined with a 2D phase velocity algorithm.

**Approach:**

An ARF-OCE system has the advantages of non-invasive detection, high resolution, high sensitivity, and high-speed imaging for quantifying the biomechanical properties of the ONH at different intraocular pressures (IOPs) and detection directions. The 2D phase velocity algorithm is used to calculate the phase velocity values at each position within the imaging region, and then the 2D velocity distribution image is realized by mapping the velocity values to the corresponding structure based on the one-to-one relationship between velocity and position. The elasticity changes can be read directly according to the quantitative relationship between Lamb wave velocity and Young’s modulus.

**Results:**

Our quantitative results show that the phase velocity and Young’s modulus of the ONH increase by 32.50% and 129.44%, respectively, with increasing IOP, which is in general agreement with the results of previous studies, but they did not produce large fluctuations with the constant change of the ONH direction. These results are consistent with the changes of elastic information in the 2D velocity distribution image.

**Conclusions:**

The results suggest that the ARF-OCE technology has great potential in detecting the biomechanical properties of the ONH at different IOPs and directions, and thus may offer the possibility of clinical applications.

## 1 Introduction

Glaucoma is an optic neuropathy, which is one of the leading causes of blindness.^1^^,^^2^ It is estimated that by 2040, ∼112 million people worldwide will be affected by glaucoma, 11 million of whom will be blind due to glaucoma.^3^ Studies have shown that a range of ophthalmic disorders encompassed by glaucoma are associated with abnormal changes in intraocular pressure (IOP), but the mechanism of interaction of elevated IOP in the development of optic neuropathy is unclear.^4^^,^^5^ Sustained elevation of IOP will cause structural changes in the posterior segment of the eye, specifically leading to outward expansion of the sclera around the optic nerve head (ONH), forcing the sieve plate and adjacent structures to undergo mechanical stresses associated with IOP, resulting in compression and remodeling.^6^ Therefore, a better understanding of the biomechanical properties of the ONH is of great clinical value to better understand how to effectively prevent and control the development of glaucomatous disease.^7^

Currently, many advanced methods are used to directly observe the ONH deformations caused by IOP changes, such as three-dimensional (3D) histomorphometry,^8^ confocal microscopy,^9^ microcomputed tomography,^10^ ultrasound images,^11^^,^^12^ magnetic resonance images,^13^ and optical coherence tomography (OCT).^14^^,^^15^ However, these methods have some limitations because the ONH and the lamina cribrosa (LC) tissues can not only displace posteriorly but also anteriorly under the influence of IOP. Therefore, it has also been observed that the LC tissues do not undergo significant displacement and deformation in a subset of patients.^16^ Therefore, there is an urgent need for an elasticity method to quantify the biomechanical properties of the ONH tissue.

Elastography is used as a non-destructive imaging tool to quantify the biomechanical properties of ocular tissues. Among the commonly used elastography techniques, magnetic resonance elastography^17^ and ultrasound elastography^18^ are the most widely used ones, which is mainly because of their high imaging depth and wide field of view, but their relatively low spatial resolution limits the detection of small changes of the velocity. Although Brillouin microscopy has higher spatial resolution and has been used to study the biomechanical properties of ocular tissues, the correlation between Brillouin frequency shift and Young’s modulus remains unclear due to the uncertainty of Poisson’s ratio, limiting its clinical application.^19^ Optical coherence elastography is an emerging elastography technique that quantifies the biomechanical properties of ocular tissues by detecting the propagation of elastic waves induced by external excitation using the OCT technique.^20^^,^^21^ Because of its micron-level resolution and sub-nanometer displacement sensitivity, the OCE technique is now widely used in ocular tissues, such as the cornea, trabecular meshwork, lens, and retina.^22^^–^^24^

In this paper, acoustic radiation force optical coherence elastography (ARF-OCE) technology is used to detect the ONH elasticity, which has been well-validated in our previous study for ocular tissue elasticity imaging.^25^^,^^26^ In this study, we first investigated the biomechanical properties of the ONH at different IOPs, meanwhile the corresponding information of the ONH at different directions have also been study since it may have an influence on the ONH elasticity, which has not been researched before. In the data processing, the phase velocity algorithm is used to calculate the phase velocity at each position of the imaging region, meanwhile the 2D velocity distribution image is realized by mapping the velocity values to the structure based on the one-to-one relationship between the velocity value and the position. Therefore, changes of the velocity and elasticity information can be directly read from the structure map based on the Lamb wave velocity-Young’s modulus relationship. Furthermore, the average Young’s modulus values of the samples are acquired.

## 2 Materials and Methods

### 2.1 Materials

To conduct ONH elasticity experiments, fresh porcine eyes (n=4) from a standard slaughterhouse are obtained, which are kept in cold saline for transfer to the laboratory. Moreover, the experiments are completed within 24 h after removal. Before the experiment, the excess tissue around the eye is removed by a scalpel to keep the ONH flush with the outer surface of the surrounding sclera. Subsequently, the entire eyes are fixed on a custom-built stent so that the cornea are facing down and the ONH are facing up. In addition, the eyeball is placed in sterile phosphate-buffered saline (PBS) to maintain the normal physiological state of the eye tissue, which also serves as a transport medium for ultrasonic wave.

### 2.2 ARF-OCE System Design

**Fig. 1:**
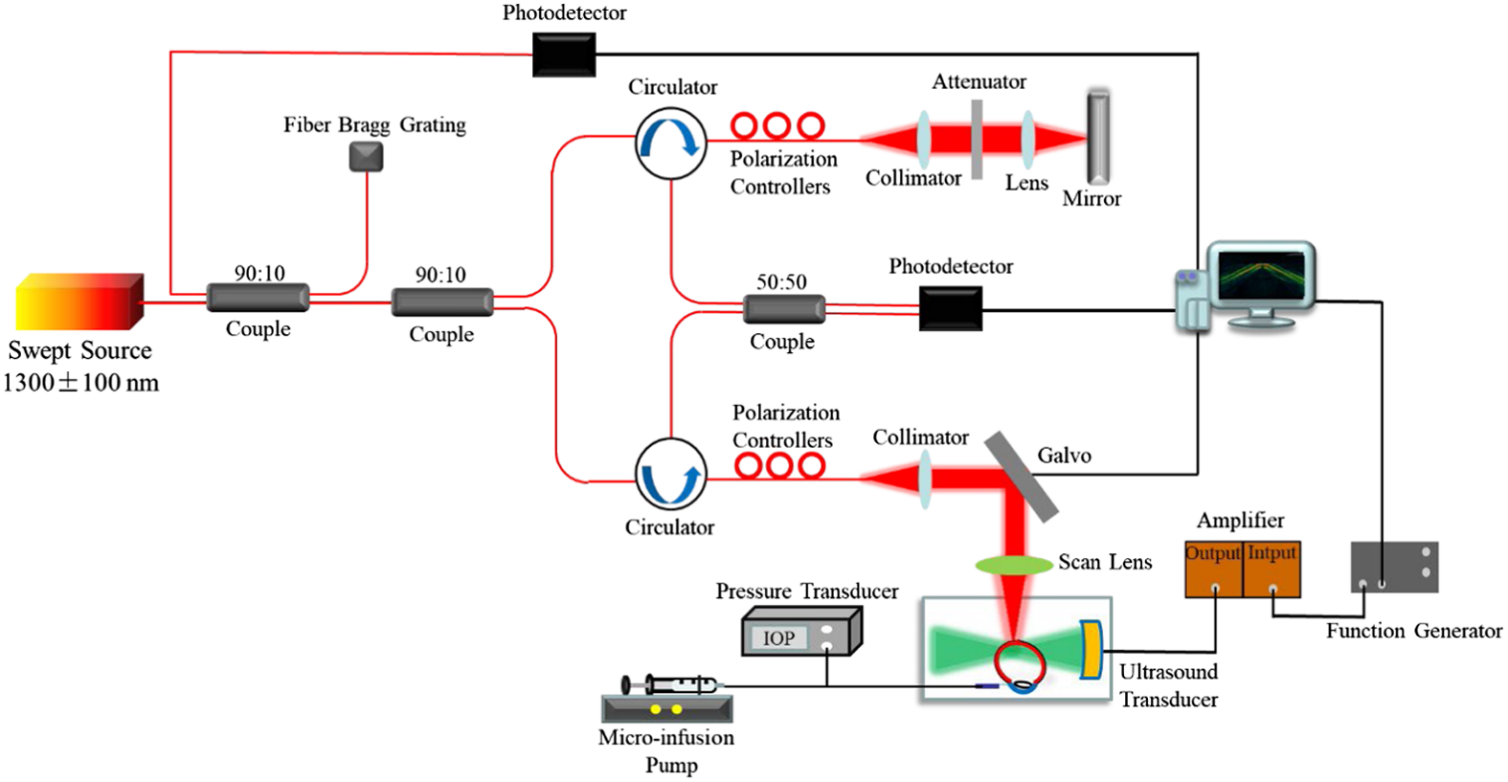
The schematic of the ARF-OCE system.

A customized ARF-OCE system is designed to assess the biomechanical properties of the ONH. The ARF-OCE system consists of a phase-sensitive OCT system and an acoustic radiation force excitation system, as shown in Fig. 1. The OCT system uses a swept-source laser (Axsun Technologies Inc., Billerica, Massachusetts) with a central wavelength of 1300 nm and a scan repetition rate of 50 kHz. The axial and lateral resolution are measured to be 6.7 and 20  μm, respectively. In order to stabilize the phase in OCT data, a fiber Bragg grating was added to produce wavelength swept trigger for the data acquisition card to record the interference signal. The optical phase stability of ARF-OCE system is calculated as ∼167.3  mrad. The light from the laser is split by the optical fiber coupler (90:10), with 90% of the output light into the sample arm and 10% output light into the reference arm. The back-reflected and back-scattered light from both arms is collected to generate an interference signal by the 50:50 coupler, which is then detected by a balanced photodetector. The signal of the detector is digitized by using a waveform digitizer acquisition card (Alazar Technologies Inc., Quebec, Canada) and processed by the computer.

The ultrasound excitation system is composed of an ultrasound transducer, a function generator, and a power amplifier. A custom-built ultrasound transducer with a center frequency of 4.5 MHz, −6  dB lateral acoustic beam width of 390  μm, and a focal length of 35 mm is used to excite tissues, which is placed at a position orthogonal to the OCT beam and can cover the OCT imaging depth in the ONH (∼0.4  mm). The trigger signal of the swept-source laser is utilized to synchronize the function generator to produce a 4.5 MHz sine wave with a duration of 500  μs, which is amplified by ∼46  dB to drive the ultrasound excitation to produce an elastic wave in the tissue.

The IOP control system mainly consists of a micro syringe pump, a catheter, a 23G needle, and a digital pressure sensor, which connects the syringe filled with electrolyte complex intraocular flush solution, the pressure sensor, and the eye through a medical T-connector. The different IOPs are controlled by adjusting the composite electrolyte intraocular rinse solution fed into the eye by the micro-infusion pump and monitored in real-time using the digital pressure sensor.

### 2.3 Data Acquisition

**Fig. 2:**
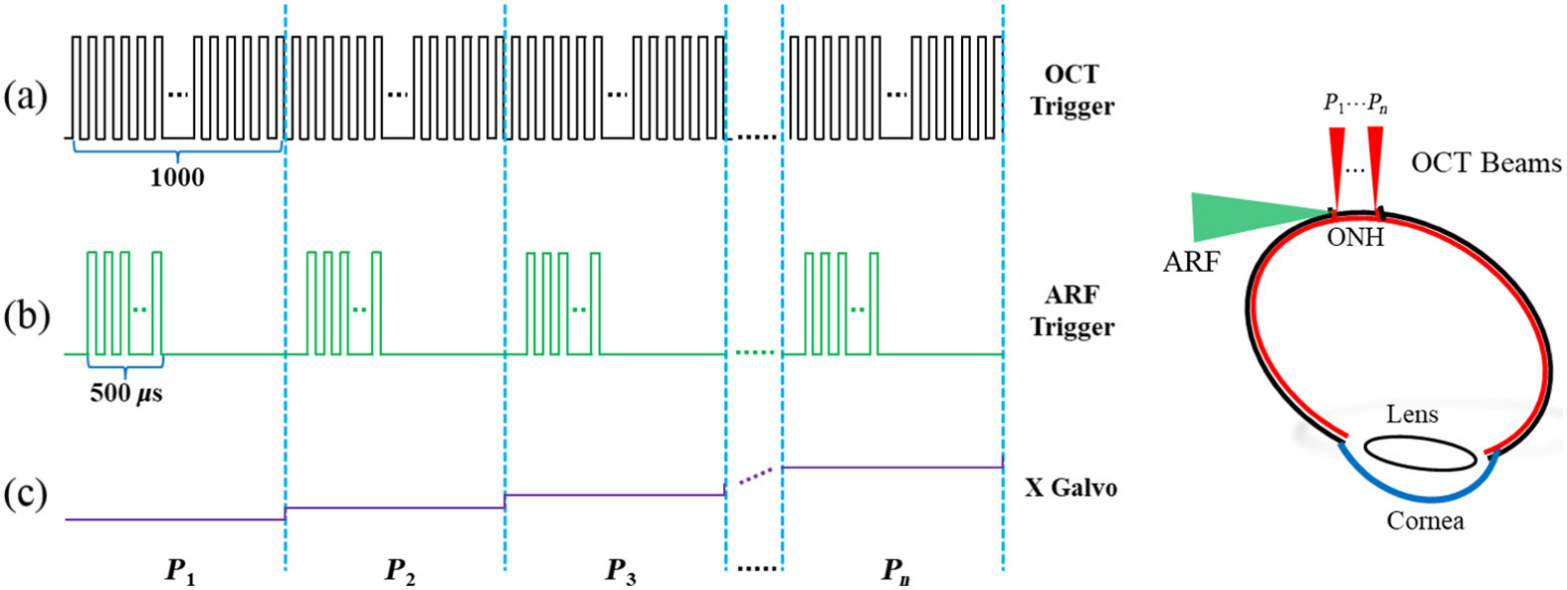
The M-B scanning protocol for the OCE system. (a) The OCT trigger signal for each M-scan image, (b) the trigger signal of the ultrasonic transducer, (c) the signals for controlling the X-axis galvanometer scanner, and (d) the position of the galvanometer in relation to the ARF.

The M-B protocol is used to visualize the propagation of elastic waves in the ONH tissue, as shown in Fig. 2, which is performed in the transverse direction from $P_{1}$ to $P_{n}$. At each lateral position, 1000 A-lines are acquired to record the intensity and phase change over time (M-mode), where the ARF excitation is generated between the 101st and 125th A-lines. After completing one M-mode data acquisition, the galvanometer scanner moves the detection beam to an adjacent position and repeats the same operation.

## 3 Results

**Fig. 3:**
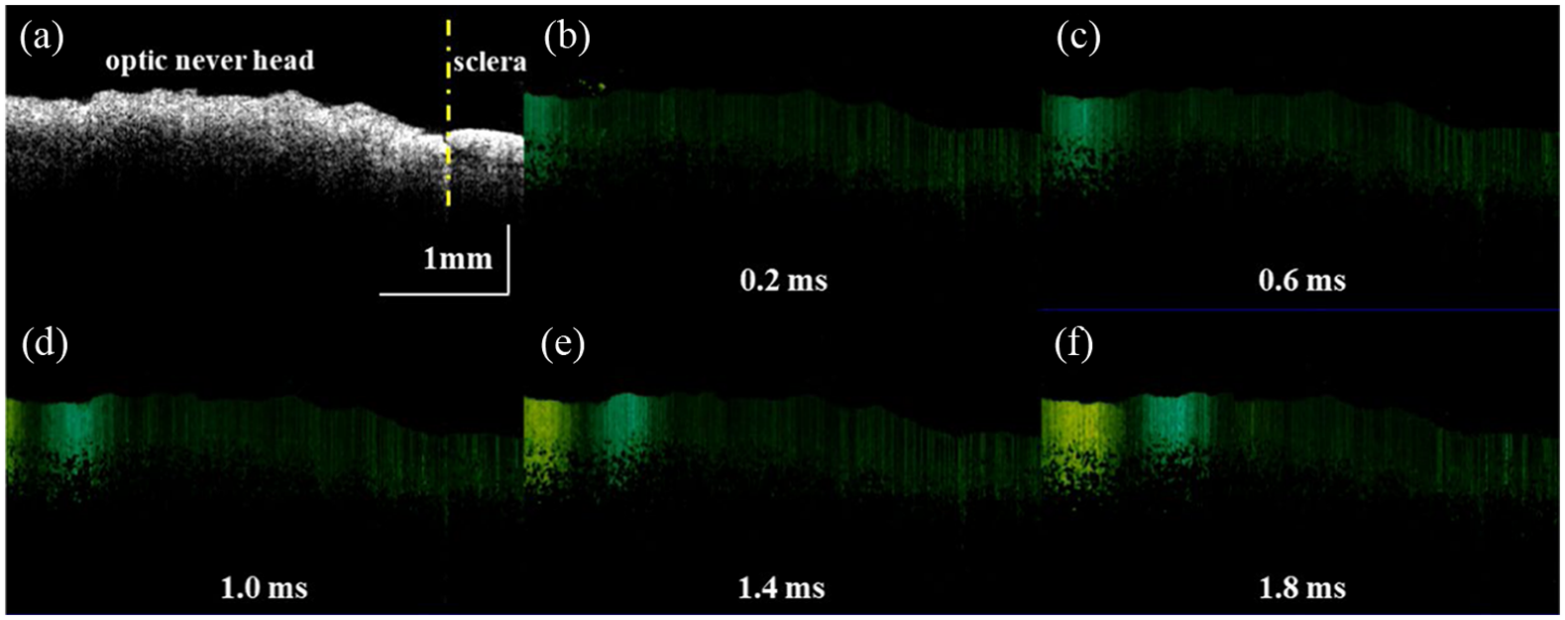
The elastography of the *ex vivo* ONH. (a) The B-scan OCT image of the ONH, (b)–(f) Doppler OCT B-scan images of the ONH at 0.2, 0.6, 1.0, 1.4, and 1.8 ms.

**Fig. 4:**
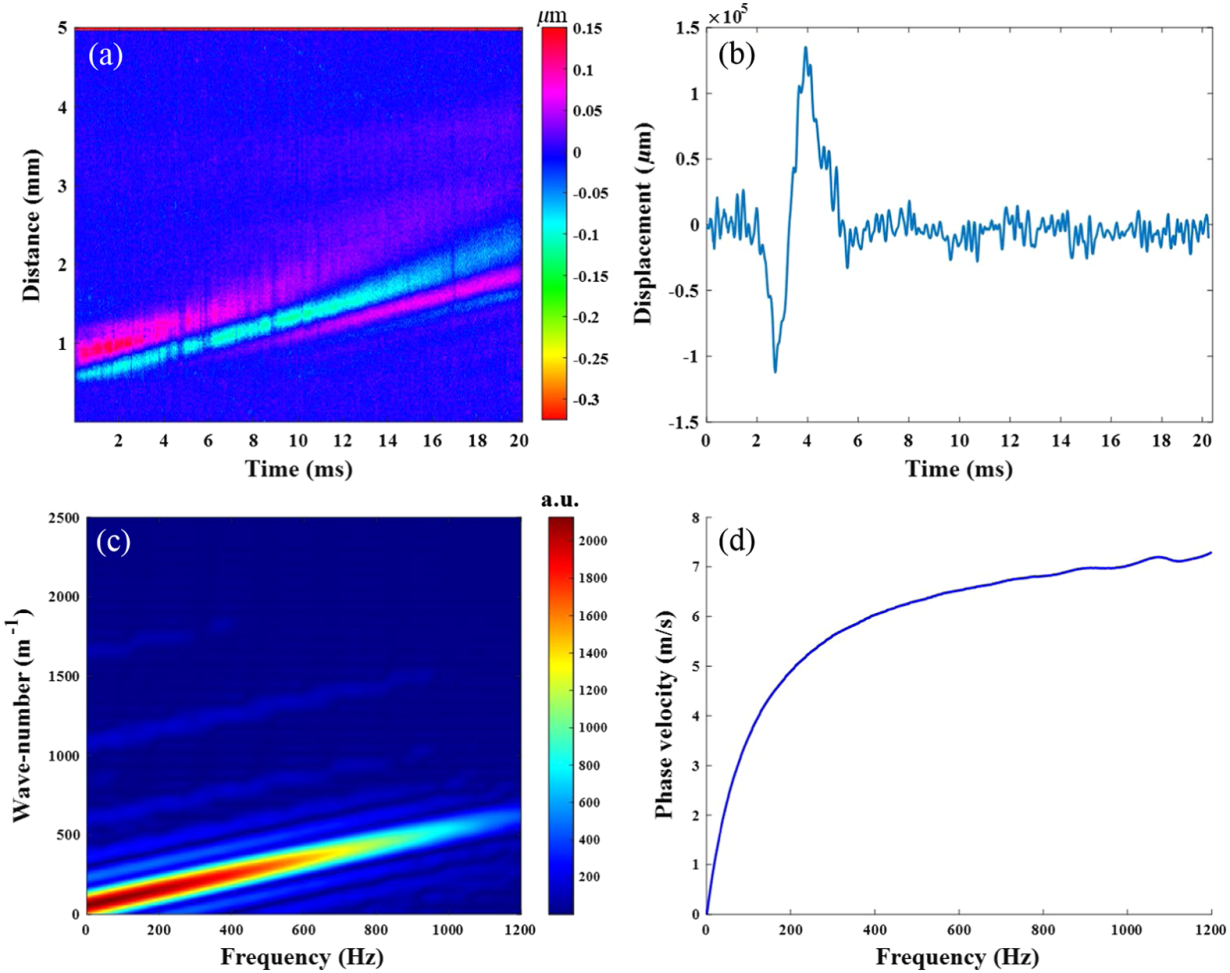
Flowchart of the elastic wave data processing. (a) Spatial–temporal displacement map, (b) vibration displacement curve, (c) wavenumber-frequency domain map, and (d) phase velocity dispersion curve.

**Fig. 5:**
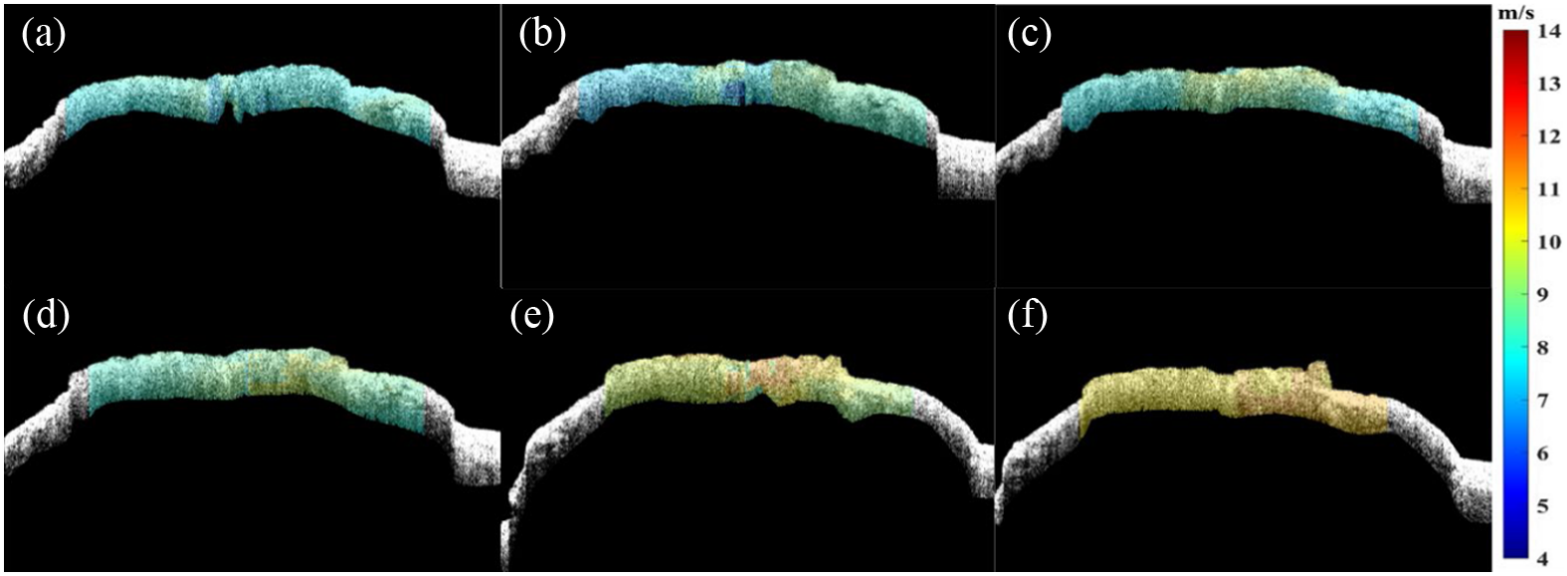
The phase velocity distribution image at different IOPs. (a) 10 mmHg, (b) 15 mmHg, (c) 20 mmHg, (d) 25 mmHg, (e) 30 mmHg, and (f) 35 mmHg.

First, we used a Lamb wave model to quantify the biomechanical properties of the *ex vivo* ONH at normal IOP (10 mmHg), as shown in Fig. 3, where the areas to the left and right of the yellow dashed line in Fig. 3(a) shows of the ONH and sclera, respectively. Figures 3(b)–3(f) show the propagation process of elastic waves at five different times. Furthermore, the calculation process of the phase velocity at a specific depth is shown in Fig. 4. The spatial–temporal maps are processed with the two-dimensional Fourier transform (2D-FFT) to obtain the k-space distribution of the frequency with respect to the wave number. According to Eq. (4), the phase velocity can be calculated by the frequency at each point divided by the maximum intensity value at that frequency. Due to the viscous characteristic of the ONH, the phase velocity profile shows a diffuse nature, as shown in Fig. 4(d). The phase velocity values in the dispersion curve are then extracted based on the main frequency corresponding to each position.^26^ Accordingly, the phase velocity values of each position in the imaging region are achieved, which are then mapped to the corresponding structure to generate the 2D velocity distribution image, as shown in Fig. 5(a), where the velocity changes can be directly observed. In addition, the change of the biomechanical properties can also be read directly from the distribution image based on the relationship between velocity and Young’s modulus, as shown in Eq. (6).

To investigate the effect of IOP changes on the biomechanical properties of the ONH, we synchronized and integrated a customized IOP control system on the ARF-OCE system, in which the IOP was controlled at 10, 15, 20, 25, 30, and 35 mmHg, respectively. Figures 5(a)–5(f) show the 2D velocity distribution images at different IOPs (color bars indicate the phase velocity values in m/s). It can be found that the phase velocity increases with the increase of the IOP values, which implies that the corresponding Young’s modulus also increases under the same condition.

**Fig. 6:**
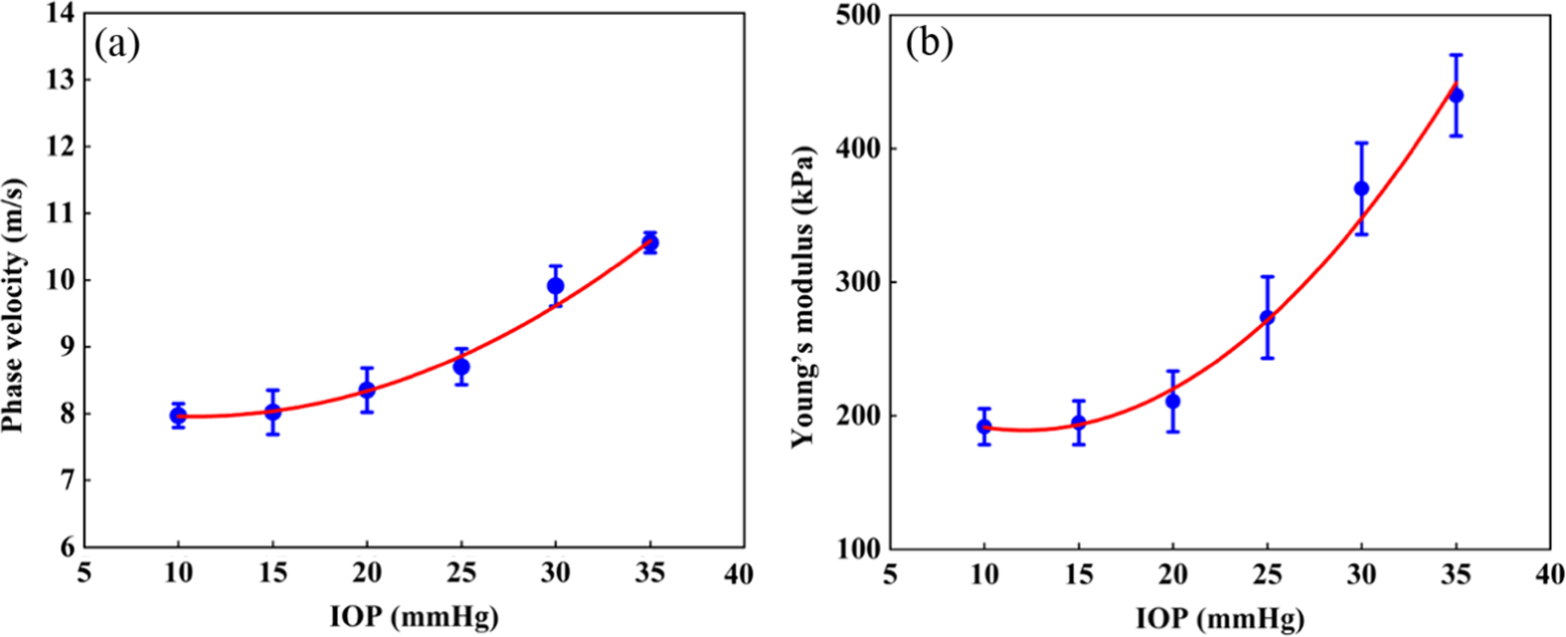
The biomechanical properties of the ONH at different IOPs. (a) Averaged phase velocity and (b) average Young’s modulus.

The mean values of the velocity under different IOPs were determined to be 7.97±0.18  m/s at 10 mmHg, 8.02±0.33  m/s at 15 mmHg, 8.35±0.33  m/s at 20 mmHg, 8.70±0.27  m/s at 25 mmHg, 9.91±0.30  m/s at 30 mmHg, and 10.56±0.15  m/s at 35 mmHg, and thus the corresponding Young’s modulus were calculated to be 191.66±13.43  kPa, 194.60±16.28  kPa, 210.61±22.71  kPa, 273.59±30.46  kPa, 369.98±34.24  kPa, and 439.76±30.39  kPa, which are shown in Table 1. In order to visualize the changing trend, the average phase velocity and Young’s modulus of the ONH at different IOPs were plotted in Fig. 6, which was fitted with a second-order polynomial function with a goodness-of-fit R-squared of 99.6% due to the wide range of tissue deformation and nonlinear effects. (Each phase velocity value, modulus value, and error bar in the figure denotes the mean and standard deviation of three repeated measurements, respectively.) It can be found from Figs. 5 and 6 that the phase velocity and Young’s modulus increase by 32.50% and 129.44%, respectively, with the increase of IOP, which is in general agreement with the results of previous studies.^27^ These results are consistent with the changes in Fig. 5.

**Table 1:** The result of *ex vivo* ONH.

IOP (mmHg)	ONH thickness (μm)	Mean phase velocity (m/s)	Elasticity (M ± SD)^a^
			Mean Young’s modulus (kPa)
10	496	7.97 ± 0.18	191.66 ± 13.43
15	530	8.02 ± 0.33	194.60 ± 16.28
20	482	8.35 ± 0.33	210.61 ± 22.71
25	560	8.70 ± 0.27	273.59 ± 30.46
30	521	9.91 ± 0.30	369.98 ± 34.24
35	530	10.56 ± 0.15	439.76 ± 30.39

a (M ± SD) represents mean and standard deviation.

**Fig. 7:**
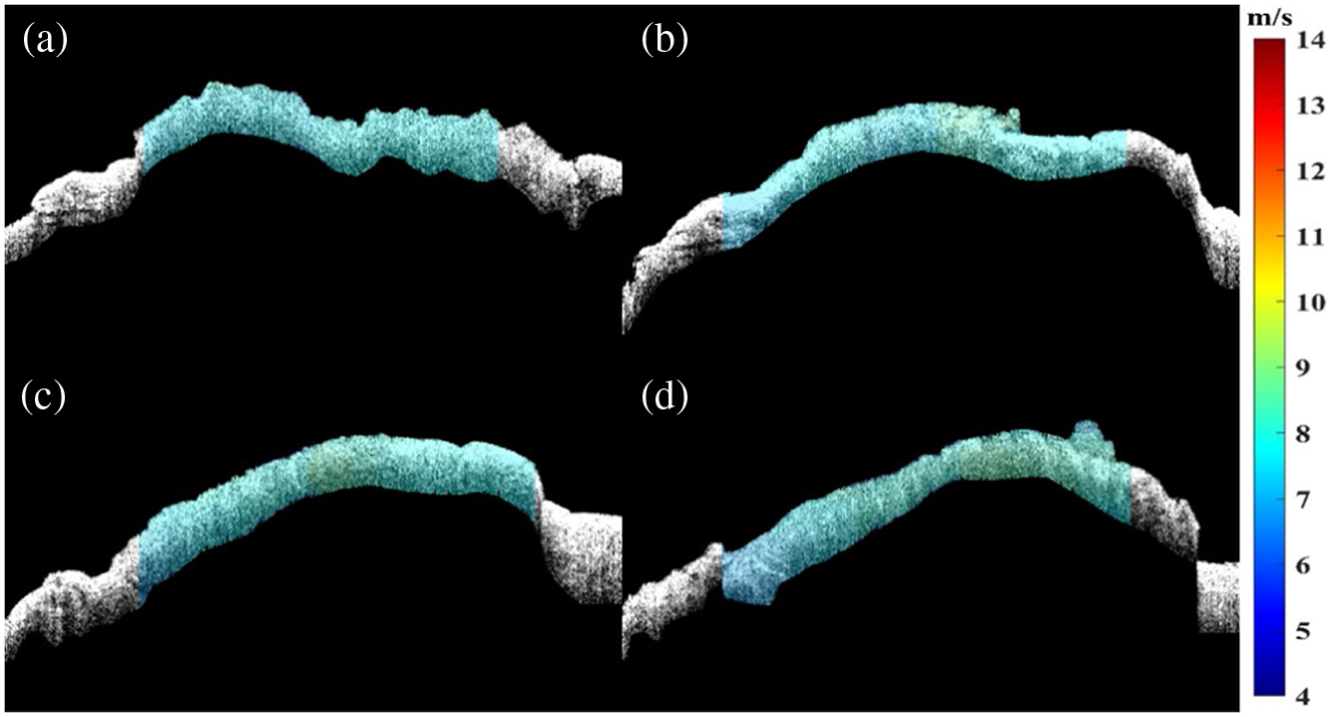
The phase velocity distribution image at different directions. (a) 0 deg, (b) 45 deg, (c) 90 deg, and (d) 135 deg.

**Fig. 8:**
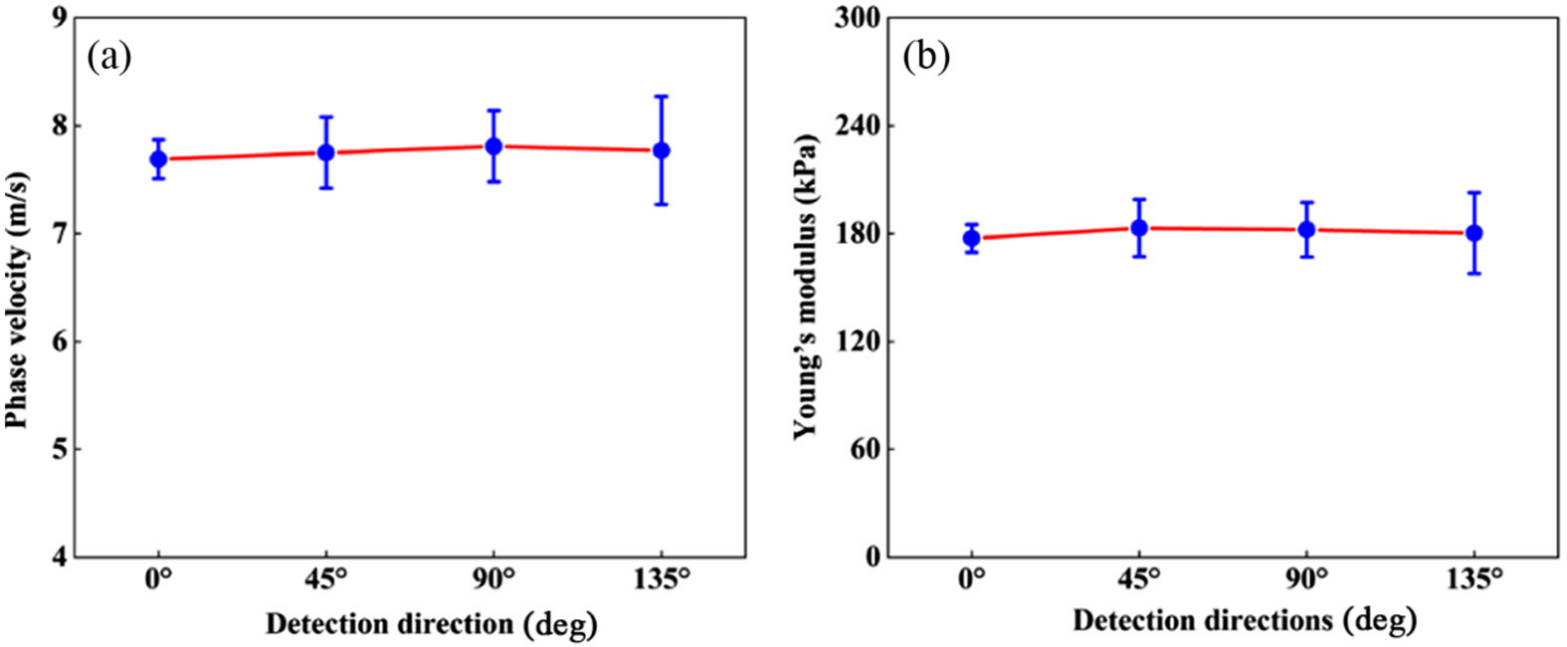
The biomechanical properties of the ONH at different directions. (a) Averaged phase velocity and (b) average Young’s modulus.

Subsequently, to better investigate the biomechanical properties of the ONH in different directions, the eye was rotated at 45 deg intervals along the center of the ONH and marked with sterile marker lines to facilitate imaging of the ONH by the system. Figures 7(a)–7(d) show the 2D phase velocity distribution obtained at different directions (color scale indicates the phase velocity values in m/s). For changing the detection direction, the mean Young’s modulus were calculated to be 177.29±7.77  kPa at 0 deg, 183.11±15.91  kPa at 45 deg, 182.14±15.20  kPa at 90 deg, and 180.24±22.46  kPa at 135 deg, which are collected in Table 2. Figure 8 plots the average phase velocity and Young’s modulus obtained at different directions. The comparison of the study results shows that the phase velocity and Young’s modulus did not produce large fluctuations with the constant change of the ONH direction.

**Table 2:** The result of *ex vivo* ONH.

Direction (deg)	ONH thickness (μm)	Mean phase velocity (m/s)	Elasticity (M±SD)^a^
			Mean Young’s modulus (kPa)
0	506	7.69 ± 0.18	177.29 ± 7.77
45	473	7.75 ± 0.33	183.11 ± 15.91
90	574	7.81 ± 0.33	182.14 ± 15.20
135	527	7.77 ± 0.50	180.24 ± 22.46

a (M ± SD) represents mean and standard deviation.

## 4 Discussion

In previous research work, several groups have investigated the biomechanical properties of the ONH by tensile test,^28^ AFM,^29^ and ultrasound elastography.^30^ However, tensile test measurements require cutting the ONH or peripapillary sclera tissue into strips meanwhile AFM measurements need micron-sized specimens.^31^^,^^32^ Therefore, the test conditions of these different research methods lead to a wide range of deviations of the obtained Young’s modulus from the *in vivo* measurements. To further quantify the biomechanical properties of the ONH and to perform non-invasive imaging of the ONH tissue. Ma et al. used a 3D cross-correlation scatter tracking algorithm to measure the tissue deformation in the posterior segment of the eye, but this method only provided stress-strain results and did not allow for quantitative measurements of Young’s modulus.^33^ Therefore, Zhou et al. developed the confocal ARF-OCE method to obtain the average elastic modulus of the ONH.^34^ Although it has demonstrated that the ARF-OCE technique can obtain the average velocity of elastic waves, the wave velocity of each position cannot be achieved and thus its 2D distribution image cannot be realized. In this work, we combined the ARF-OCE system with the phase velocity algorithm to generate 2D phase velocity distribution images of the ONH at different IOPs and detection directions. Our method not only can obtain the average Young’s modulus but also can offer the possibility to read the elasticity changes directly from the structure map. Therefore, this study further demonstrated the superiority of the ARF-OCE system in detecting the biomechanical properties of the ONH.

Although the technique has been used to quantitatively analyze the biomechanical properties of the ONH with elevated IOP and altered detection direction, there are still some challenges and limitations that need to be addressed for the successful translation of the technology for clinical applications. First, faster lasers are required to reduce the impact of artifacts caused by body shaking, breathing, and heartbeat on the imaging quality.^35^ In addition, due to the faster propagation of elastic waves in stiffer objects, the wave propagation may not be tracked if the acquisition speed of the imaging system is not fast enough.^36^ Second, PBS was used as the ultrasonic propagation medium; however, it will inevitably lead to a bad user experience and thus an air-coupled ultrasound transducer should be employed to better fulfil requirements of *in vivo* measurements. Meanwhile, developing a novel OCE system based on a broadband laser with a central wavelength of 850 nm and a zoom ring transducer will be used to detect ONH in the retina through the lens. In addition, the mechanical index (MI) of the ARF applied in our study should be well below the FDA ophthalmic MI standard of 0.23.^37^ Third, the effective depth resolution of the system can be improved by increasing the sampling points in the depth direction. Finally, a detailed analysis of wave propagation models in bounded media is essential to assess tissue biomechanical properties. In future studies, more sophisticated and advanced wave propagation models will be developed to quantify the viscoelasticity of tissues by evaluating the dispersion of elastic wave propagation.

## 5 Conclusion

In this paper, we have demonstrated that the ARF-OCE technology can be used to quantify the biomechanical properties of the ONH at different IOPs and detection directions. More importantly, the 2D velocity distribution images can be realized based on the one-to-one correspondence between the velocity value and the position, where changes of the velocity and Young’s modulus can be directly read. Moreover, our quantitative result shows that the Young’s modulus of the ONH increases significantly with increasing IOP, but it basically does not change with the change of the ONH detection direction. As a result, this technology has great potential in detecting the biomechanical properties of the ONH at different IOPs and directions, and thus may offer the possibility of clinical applications.

## 6 Appendix: Data Processing

### 6.1 Displacement Map

In our experiments, the phase-resolved Doppler algorithm was adopted to extract the phase information that varies with time. With the M-mode OCT image, Doppler phase shift profile of the adjacent A-lines can be calculated using the following equation:^38^

Δφ=tan−1[Im(Fm×Fm+1*)Re(Fm×Fm+1*)], (1)

where $F_{m}$ is the complex signal at the given position of the M-mode, $F_{m+1}$ is $F_{m}$ at the next time point, and $F^{*}$ is the conjugate complex of F. Im () and Re () are the imaginary and real parts of the OCT complex signal, respectively. Thus, according to phase change Δφ, the axial displacement Δd can be calculated by using the following equation:^27^

$$\Delta d=\frac{\lambda_{0}}{4\pi n}\Delta \varphi ,$$ (2)

where $\lambda_{0}$ is the center wavelength of the laser source, and n is the refractive index.

### 6.2 Velocity and Modulus Calculation

Since ONH is a thin plate structure at the tissue structure level and is small compared to the transverse wave wavelength. Lamb waves usually propagate in a medium with upper and lower boundaries, thus the lamb wave is identified as the elastic waves propagating in the ONH. The lamb wave can only propagate in the low frequency range due to the viscosity in soft tissues. Moreover, the zeroth order mode can travel at any frequency and is most commonly detected in the low-frequency range as compared with non-zeroth order mode Lamb waves. For a thin plate immersed in an incompressible fluid, the dispersion equation based on the zero-order antisymmetric Lamb wave mode is^39^

$$4k_{L}^{2}\eta \beta \;\cosh(\eta h)\sinh(\beta h)-(2k_{L}^{2}-k_{S}^{2})\sinh(\eta h)\cosh(\beta h)=k_{S}^{4}\;\cosh(\eta h)\cosh\beta h,$$ (3)

where $\beta =\sqrt{k_{L}^{2}-k_{S}^{2}}$, $k_{L}=\omega /c_{L}$ is the Lamb wavenumber, ω is the angular frequency, $c_{L}$ is the frequency dependent phase velocity, $k_{S}=\omega \sqrt{\rho /U}$ is the shear wavenumber, U=μ+iωη is the shear modulus, μ and η are shear elasticity and shear viscosity, respectively, and h is the half thickness of the ONH.

The spatial–temporal Doppler displacement map of the entire ONH tissue is first obtained from the intensity and phase of the raw data. Due to the presence of the low-frequency noise, the spatial–temporal Doppler displacement map is filtered by a high-pass filtering algorithm and then converted to the wavenumber/frequency domain (k-space map) by 2D-FFT. Based on the wavenumber-frequency domain map, the phase velocity of the Lamb wave is obtained by dividing the frequency by the corresponding maximum wavenumber as follows:^40^

$$c_{L}=\frac{\omega }{k_{L}}=\frac{2\pi f}{k_{L}},$$ (4)

where ω is the angular frequency, and $\kappa_{L}$ is the Lamb wave number. Since the thickness of the ONH tissue is much smaller than the wavelength of the elastic wave generated by the excitation, the Lamb wave velocity $V_{L}$ in the vacuum can be expressed as^41^

$$V_{L\_\text{vacuum}}=\sqrt{\frac{2\pi \times f\times h\times V_{S}}{\sqrt{3}}},$$ (5)

where f is the frequency, h is the ONH thickness, and $V_{s}$ is the shear wave velocity. Since the ONH tissue is submerged by liquid, the corresponding Lamb wave velocity can be corrected by multiplying a factor of $1/\sqrt{2}$ the total longitudinal wave leakage and the total transverse wave reflection at both boundaries ($V_{L}=V_{L\_\text{vacuum}}/\sqrt{2}$). According to $E=3\rho V^{2}$, the corresponding Young’s modulus can be calculated based on the Lamb wave velocity by the following equation:^42^

$$E=\frac{9\rho \times V_{L}^{4}}{{\left(\pi \times f\times h\right)}^{2}},$$ (6)

where ρ is the density of the ONH, $V_{L}$ is the Lamb wave velocity.

## Data Availability

The processing of the code used in this paper has been published in a related literature report,^26^ which contains only the development of the code. The detailed code is available from the respective authors upon reasonable request.
